# Supplementary material for: First and second wave dynamics of emergency department utilization during the COVID-19 pandemic: A retrospective study in 3 hospitals in The Netherlands
Source: PLoS One. 2023 Feb 16;18(2):e0279105. doi: 10.1371/journal.pone.0279105 (PMC9934309; doi:10.1371/journal.pone.0279105)
Supplement: S1 File — (DOCX) [file pone.0279105.s001.docx]

# Title of Dataset: First and second wave dynamics of emergency department utilization during the COVID-19 pandemic: a retrospective study in 3 hospitals in the Netherlands

---

During certain phases of the COVID-19 pandemic, a decrease was observed in emergency department (ED) utilization. Although this phenomenon has been thoroughly characterized for the first wave (FW), second wave (SW) studies are limited. We examined the changes in ED utilization between the FW and SW, compared to 2019 reference periods. We performed a retrospective analysis of ED utilization in 3 Dutch hospitals in 2020. The FW and SW (March-June and September–December, respectively) were compared to the reference periods in 2019. ED visits were labeled as (non-)COVID-suspected. Further details can be read in our publication in PLOS ONE.

## Description of the Data and file structure

All data were automatically substracted from patient records. The first variables contain age, sex and COVID-19 suspicion. The next 5 variables are the ED utilization related variables. The destination after ED variable has information on whether patients were admitted or what other possible destinations after ED visit were. The last variables in the dataset contain filters to select the wave or comparison group of interest. The only omitted variables in this dataset are the variables indicating from which of the participating hospital the data originated and what the date of ED visit, in order to be an anonymous dataset.

## Sharing/access Information

Please contact the authors when using or downloading the data.
