# Supplementary material for: First and second wave dynamics of emergency department utilization during the COVID-19 pandemic: A retrospective study in 3 hospitals in The Netherlands
Source: PLoS One. 2023 Feb 16;18(2):e0279105. doi: 10.1371/journal.pone.0279105 (PMC9934309; doi:10.1371/journal.pone.0279105)
Supplement: Attachment — Submitted filename: Dear editor.doc [file pone.0279105.s003.doc]

Dear editor,

Thank you for the opportunity to review this manuscript.

1- Please mention the weak and strong points of your study
2- The authors do discuss the specifics of the hospital involved in the study regarding comparison of annual admission in the ED with the past COVID-19 period
3- Literature review is so incomplete. Please discuss the following article in the discussion:

Bagi HM, Soleimanpour M, Abdollahi F, Soleimanpour H. Evaluation of clinical outcomes of patients with mild symptoms of coronavirus disease 2019 (COVID-19) discharged from the emergency department. PLoS One. 2021 Oct 21;16(10):e0258697. doi: 10.1371/journal.pone.0258697

4- Discussion should be improved.
5- How did the authors delete the effect of confounding factors from the study outcome?
6- What is the new finding of this study compared to previous ones this field?
7- How did the authors delete the effect of confounding factors from the study outcome?
